# ZnO nanorod array-coated mesh film for the separation of water and oil

**DOI:** 10.1186/1556-276X-8-183

**Published:** 2013-04-20

**Authors:** Hong Li, Yushan Li, Qinzhuang Liu

**Affiliations:** 1 School of Physics and Electronic Information, Huaibei Normal University, Huaibei 235000, People’s Republic of China; 2 Department of Physics, Heze University, Heze 274015, People’s Republic of China

**Keywords:** Superhydrophobicity, Superhydrophilicity, ZnO nanorod arrays

## Abstract

Dense and vertically aligned ZnO nanorod arrays with a large area have been fabricated successfully on the stainless steel mesh by a simple chemical vapor deposition method. The coated mesh exhibited both superoleophilic and superhydrophobic properties, even if it was not modified by low surface energy materials. The separation efficiencies were more than 97% in the filtration of water and oil. Besides, the wettability of the coated mesh was still stable after it was soaked in the corrosive solutions for 1 h. A detailed investigation showed that the coated mesh has the best superhydrophobic property when the stainless steel mesh pore size was about 75 μm.

## Background

With the development of economy and society, the oil pollution has become a worldwide challenge due to its serious threat to people’s livelihoods and the ecological environment [1-4]. Therefore, the removal of oil from water is becoming imperative. Many methods were employed to solve the oil pollution, such as chemical dispersant [5], in situ burning [6], and oil-absorbing materials [7-9]. However, these methods usually have some drawbacks, including low separation efficiency, poor recyclability, and high operation costs. In order to overcome these problems, the solid surfaces with both superoleophilicity and superhydrophobicity have incited broad attention due to the application in the separation of oil and water [10].

The wettability of the solid surface is a very important property, and it can be regulated by surface free energy and surface structure [11-15]. The superhydrophobic surfaces were usually achieved by modifying rough surfaces with low-surface energy materials [16]. The filtration of water and oil has been achieved using the stainless steel mesh modified through polytetrafluoroethylene [10]. Wang et al. [17] have fabricated successfully the copper filter which can be used in the filtration of water and oil by grafting hexadecanethiol. However, the organic matters which were used in chemical modification are usually expensive and harmful. In addition, they were easily removed from the surface due to their solubility in oil.

In this paper, ordered ZnO nanorod arrays have been fabricated successfully on the stainless steel mesh by a simple chemical vapor deposition method. The superhydrophobic and superoleophilic mesh could separate water from oil effectively, and its wettability kept stable even if it was soaked in the corrosive solutions for 1 h. The coated mesh will have a potential application in oil spill cleanups.

## Methods

The ZnO nanorod arrays which were coated on the surface of the stainless steel mesh were synthesized via a chemical vapor deposition process. A piece of stainless steel mesh whose pore size was 75 μm and whose surface area was about 1 × 5 cm^2^ was cleaned by being soaked in acetone for 20 min, and then, ultrasonic cleaning was done for 15 min. After being rinsed with deionized water, they were soaked in ethanol for 30 min, rinsed with deionized water again, and dried in the oven at 50°C for 30 min. Then, an Au film whose thickness was about 50 nm was deposited on the substrate. High-purity Zn powders (99.999%) were placed in the quartz boat, and then, the quartz boat was put in the center of the tube furnace. The substrate was placed about 5 cm away from the quartz boat. Previous to the growth, the tube furnace was pumped to 5 Pa. Subsequently, the temperature of tube furnace was raised to 650°C for 30 min under the protection of Ar (120 sccm). Then, O_2_ (80 sccm) was introduced into the furnace. The growth lasted for 40 min. Then, the whose system was cooled to 25°C. After that, the ZnO nanorod arrays were grown on the surface of the stainless steel mesh. Lastly, the as-prepared sample was stored in the dark room for 2 weeks before it was measured.

The surface morphology of the ZnO nanorod was studied using scanning electron microscope (SEM, Hitachi S4700, Chiyoda-ku, Japan). The phase identification of the ZnO nanorod was carried out with X-ray diffraction (XRD, Cu Kα). The contact angles on the as-grown sample were measured by contact angle meter (DSA100, KRÜSS, Hamburg, Germany).

## Results and discussion

**Figure 1:**
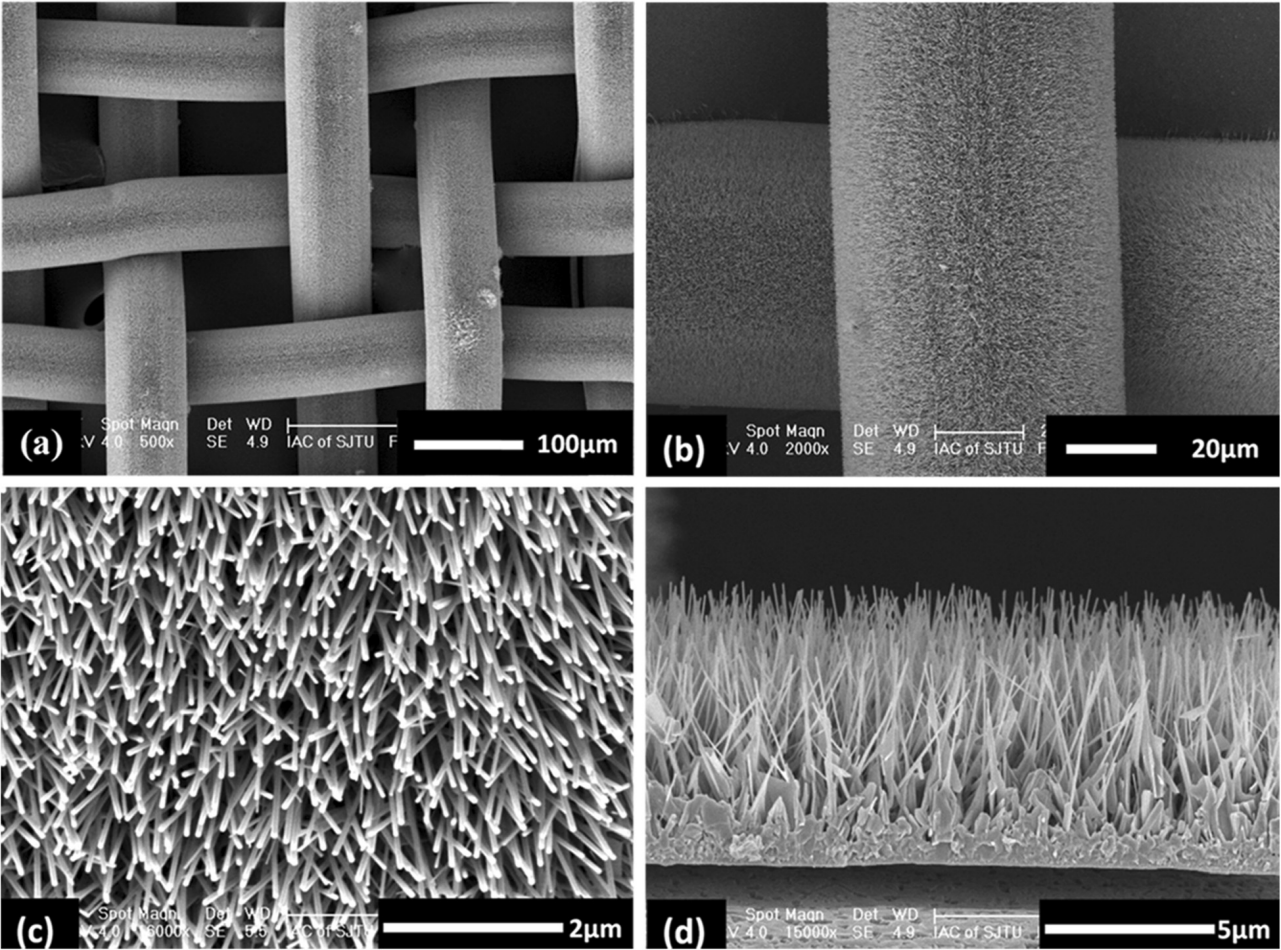
**SEM images of the as-grown ZnO nanorod arrays on the stainless steel mesh. (a)** Large-area view of the coated mesh, **(b)** top images of the ZnO nanorod arrays on a stainless steel wire, **(c)** high-magnification ZnO nanorod arrays on a stainless steel wire, and **(d)** SEM side views of the ZnO nanorod arrays with height about 4 μm.

**Figure 2:**
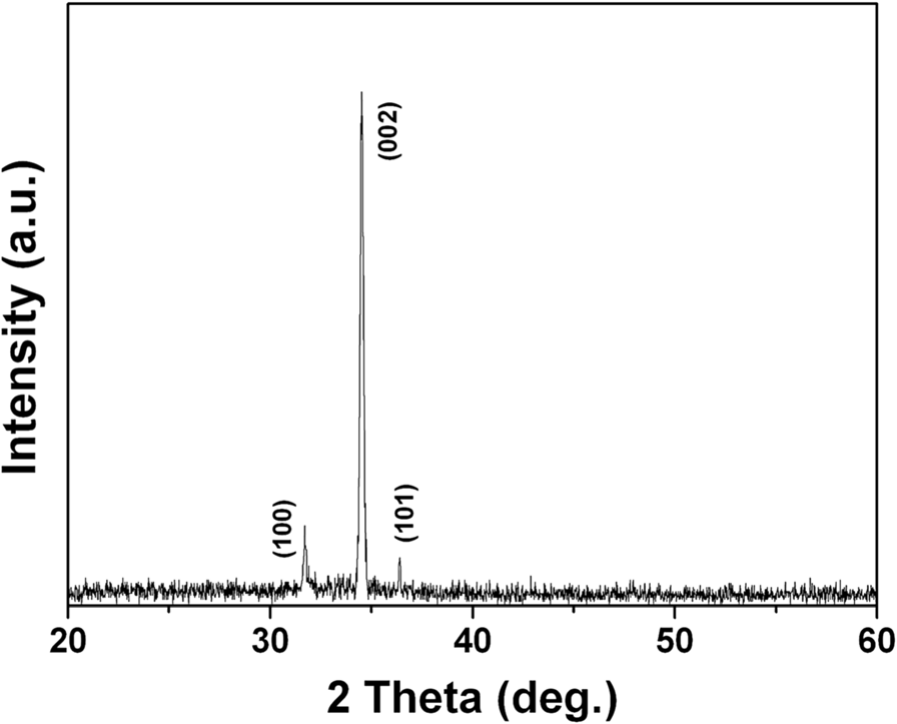
XRD patterns of the as-grown sample.

Figure 1 indicates the SEM images of the as-grown sample. As shown in Figure 1a, the surface of stainless steel mesh was covered uniformly with the ZnO nanorod arrays. It can be found that the highly uniform and densely packed ZnO nanorods were grown on a stainless steel wire; the average diameter of the ZnO nanorod is about 85 nm (Figure 1b,c). Figure 1d shows the cross-sectional view of the ZnO nanorod arrays. We can found that the ZnO nanorod arrays are almost vertical to the surface of the stainless steel wire, and the heights are about 4 μm. Figure 2 shows the XRD pattern of the ZnO nanorod arrays coated on stainless steel mesh. Three peaks (100), (002), and (101) can be deduced. The intensities of (100) and (101) peaks are much lower than the (002) peak. This indicates that the as-grown sample is a polycrystalline wurtzite ZnO and along [001] direction.

**Figure 3:**
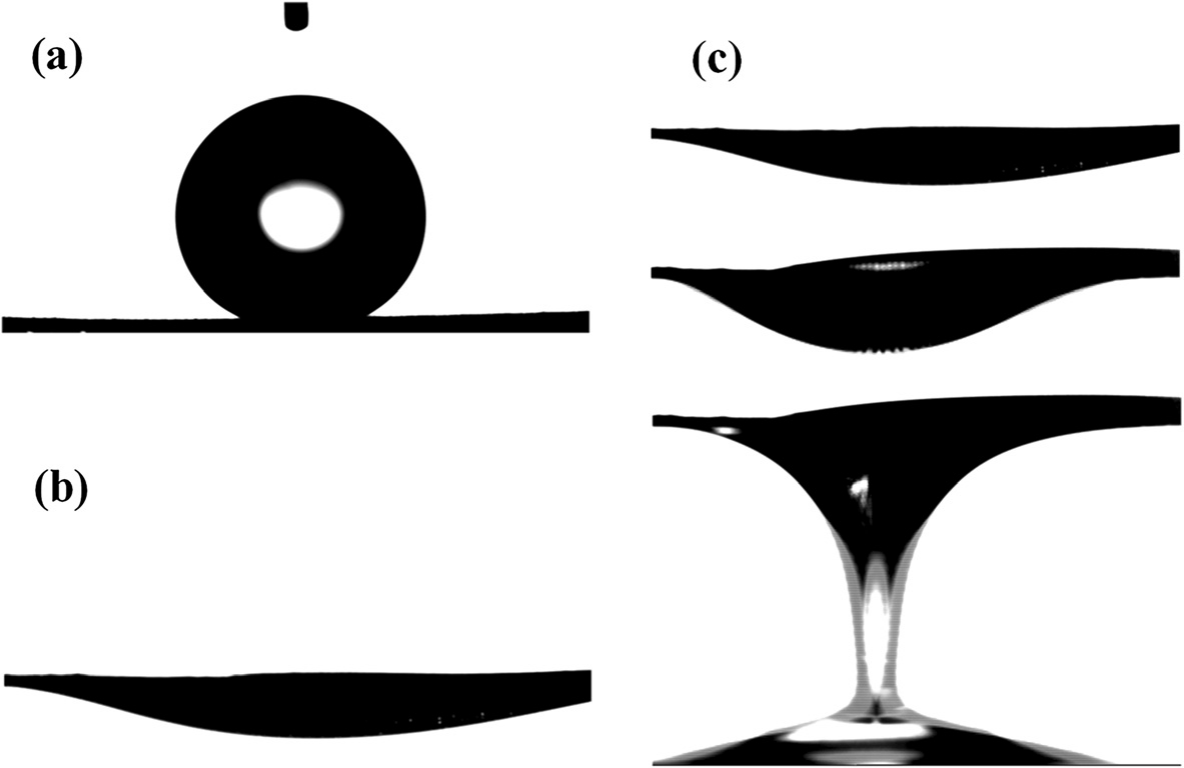
**The shape of water and oil droplet on the as-prepared mesh film. (a)** Water contact angle about 157 ± 1°, **(b)** oil contact angle about 0°, and **(c)** permeating behavior of oil on the mesh film.

The slow-growing planes usually have low surface free energy [18]. The growth rates of the ZnO crystal were reported to be [−100] > [−101] > [001] ≈ [00–1] [19]. Figure 2 shows that the surface of the ZnO nanorod is the (001) plane. So, the surface free energy of the as-grown ZnO nanorod arrays is lowest compared to the other orientations. Figure 1 also shows that the coated mesh has the rough surface. Such hierarchical micro/nanostructure ZnO nanorods array can trap enough air in between substrate surface and water droplet. Therefore, the coated mesh is expected to show superhydrophobicity. The wettability of the as-grown sample was evaluated via the water contact angle (WCA). Figure 3a presents that the WCA on the as-grown sample is about 157 ± 1°, which indicates that the coated mesh is superhydrophobic.

**Figure 4:**
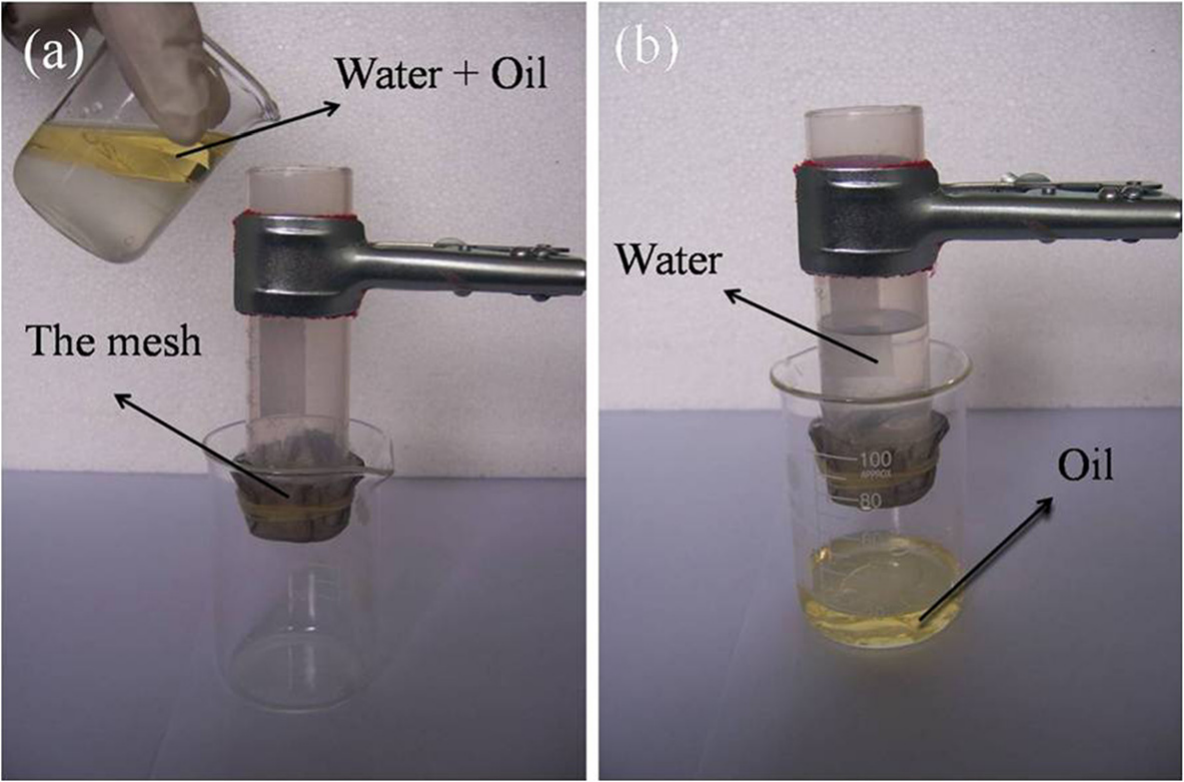
**Concrete experimental process of separation oil and water. (a)** Before separation. **(b)** After separation.

According to the Wenzel equation [20], the oleophilicity of the oleophilic materials can be enhanced via increasing the roughness of the sample surface. The coated mesh is expected to show superoleophilicity because of the hierarchical micro/nanostructure ZnO nanorods array on the oleophilic stainless steel mesh. Figure 3b shows that the oil contact angle (OCA) on the as-grown film is about 0°, and the oil droplet will penetrate freely through the coated mesh (Figure 3c). In order to confirm the feasibility of the coated mesh in practice, as shown in Figure 4, the mixtures of diesel oil and water (volume ratio 3:7) were slowly poured into the test tube; the oil permeated freely through the coated mesh and flowed into the beaker, while the water was repelled on the filter.

**Figure 5:**
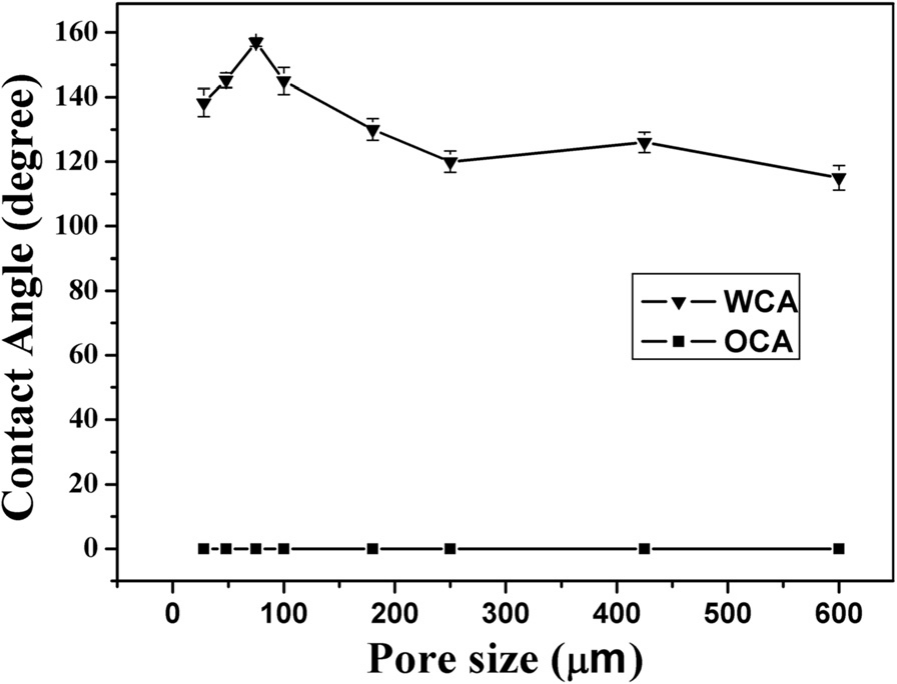
**Relationship between the pore size of the original stainless steel mesh and the contact angles.** Of water and oil on the corresponding coating film.

It has been reported that the pore sizes of the original stainless steel mesh are critically important to the wettability of the coated mesh [10]. Figure 5 shows the dependence of WCAs and the OCAs on the pore sizes of the original stainless steel mesh. The WCAs on the coated mesh increase with the increase of the pore sizes and have maximum value when the pore size is about 75 μm. Then, the WCAs became smaller when the pore sizes increase further. The OCAs are always kept at 0° and do not change with the change of the pore sizes. It is generally considered that the larger the WCAs and OCAs distinction, the easier the filtration of water and oil. It can be shown that 75 μm is the optimum pore size for the filtration of water/oil mixtures.

**Figure 6:**
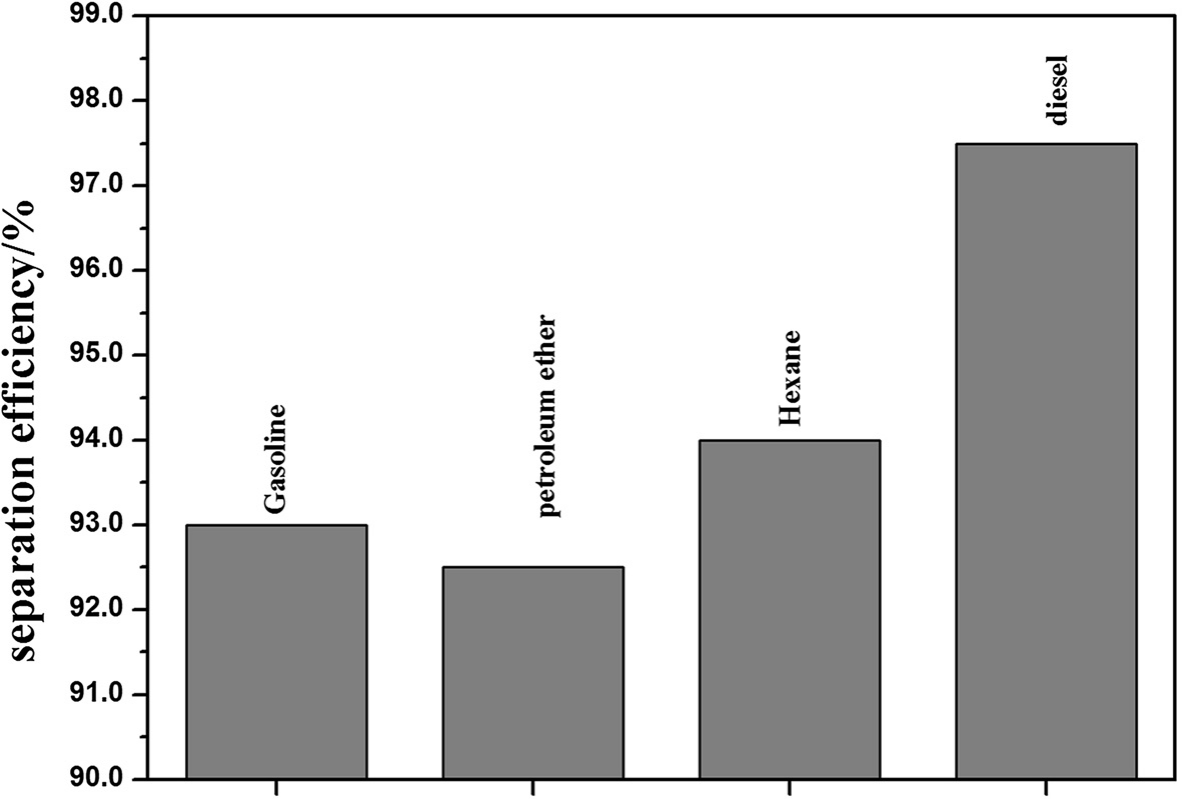
The separation efficiency of the as-prepared mesh film for different mixtures of oil and water.

The separation efficiency of the as-grown sample was studied by oil rejection coefficient (*R* %) [21].

(1) $$R\left(\%\right)=\left(1-\frac{C_{p}}{C_{0}}\right)\times 100$$

where *C*_0_ is the oil concentration before filtration and *C*_*p*_ is the oil concentration after filtration. Hexane, diesel oil, petroleum ether, and gasoline water/oil mixtures were used in the process of experiment. The specific separation efficiency is shown in Figure 6. The separation efficiency for the mixture of the diesel and water has the maximum value above 97%.

**Figure 7:**
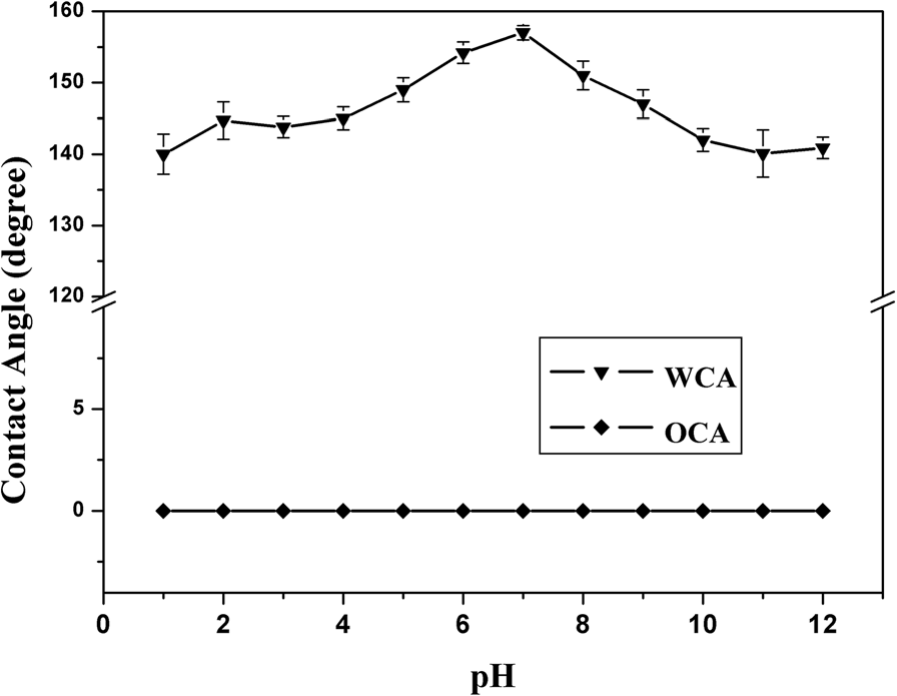
Relationship between pH values and contact angles of water and oil on corresponding coating film.

Usually, the wettability is sensitive to the environment. In order to study the stability of the as-grown sample, the coated meshes with pore size of 75 μm were dipped into the corrosive solutions in which the pH is between 2 and 13 for 1 h. The diagram shows that the mesh is still hydrophobic and superoleophilic, as shown in Figure 7. The results show that the wettability of the coated mesh is stable, which indicate that the coated mesh is an attractive material for the filtration of water/oil mixtures.

**Figure 8:**
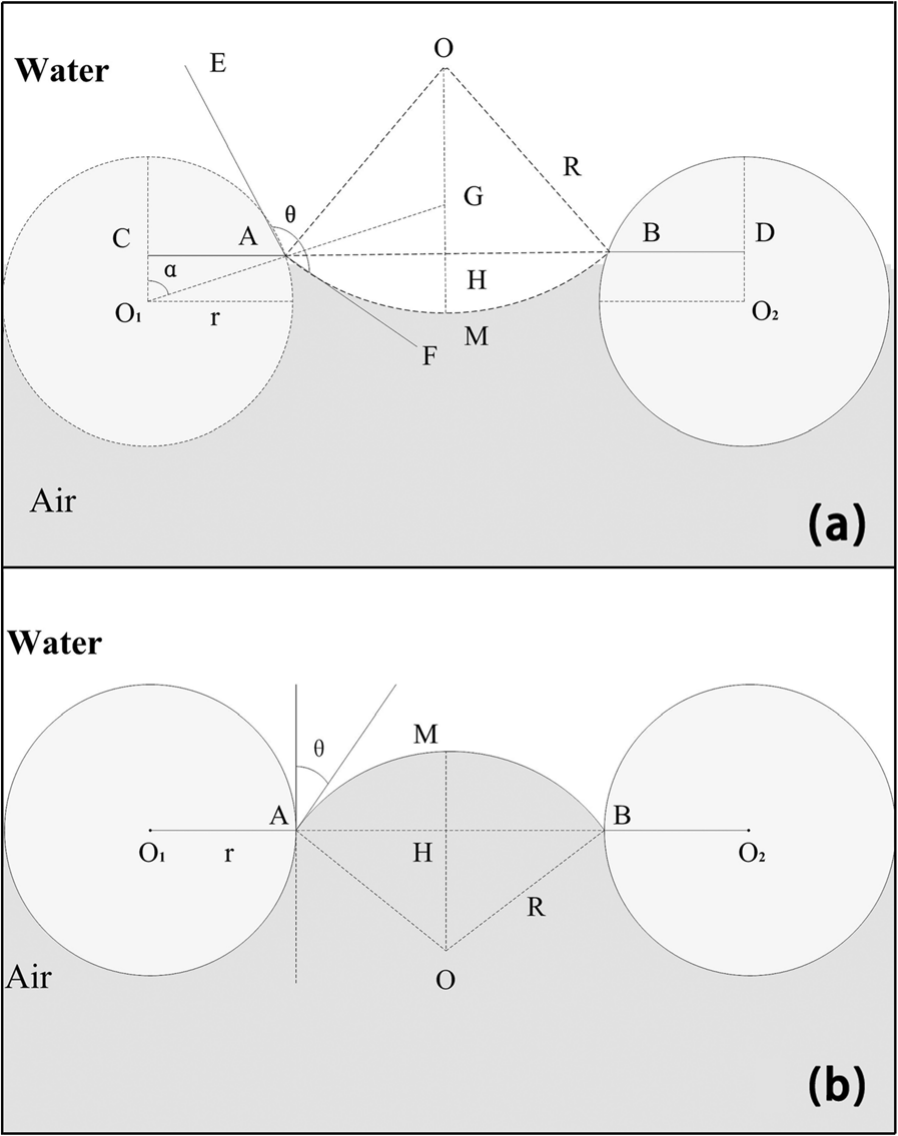
**Schematic diagrams of the wetting model of the mesh film coated by ZnO nanorod arrays. (a)**The coated mesh is impermeable to water, and **(b)** the coated shows good oil permeability. O is the center of the spherical cap of the meniscus; O_1_ and O_2_ are the cross section center of the mesh.

To further understand the mechanism that the water and oil have different contact angles on the coated mesh, the process was modeled in Figure 8.

(2) $$\Delta P=\frac{2\gamma_{L}}{R}=-l\gamma_{L}\left(\cos\theta_{A}\right)/A$$

All the parameters refer to reference [16]. The coated mesh shows superhydrophobicity due to the lower surface free energy and the higher surface roughness. It can be shown from Figure 8a and Equation 2 that the Δ*P* > 0 when *θ* > 90°. So, the water cannot penetrate the coated mesh. From Figure 8b and Equation 2, we can see that Δ*P* < 0 when *θ* < 90°, so the coated mesh cannot sustain a little oil, and good penetration can be achieved. In addition, it can also be seen from Equation 2 that the oil which has the larger surface tension will penetrate the coated mesh easier. So the water/diesel oil mixture has the maximum value, which is in accord with our experimental result.

## Conclusions

In this paper, high-quality ZnO nanorod arrays were achieved by chemical vapor deposition route on the stainless steel mesh. The coated mesh exhibited superhydrophobic properties due to the special micro/nanoscale hierarchical ZnO nanorod arrays and the highly *c*-axis-oriented crystal. At the same time, the coated mesh was superoleophilic, and the stability of the wettability was also good. So, the coated mesh can filter water/oil mixtures, and the separation efficiencies were more than 97%. In addition, the effect of different pore sizes of the original stainless steel mesh on the superhydrophobicity and superoleophilicity of the coated mesh was studied. The coated mesh promises a potential application for the water/oil separation.

## Abbreviations

OCA: Oil contact angle; SEM: Scanning electron microscope; WCA: Water contact angle; XRD: X-ray diffraction.

## Competing interests

The authors declare that they have no competing interests.

## Authors’ contributions

HL participated in the design of the study, carried out the experiments, performed the statistical analysis, and drafted the manuscript. YSL participated in the design of the study. QZL revised the manuscript. All authors read and approved the final manuscript.
